# Reversal Of Arterial Disease by modulating Magnesium and Phosphate (ROADMAP-study): rationale and design of a randomized controlled trial assessing the effects of magnesium citrate supplementation and phosphate-binding therapy on arterial stiffness in moderate chronic kidney disease

**DOI:** 10.1186/s13063-022-06562-9

**Published:** 2022-09-12

**Authors:** Emma A. Vermeulen, Coby Eelderink, Tiny Hoekstra, Adriana J. van Ballegooijen, Pieter Raijmakers, Joline W. Beulens, Martin H. de Borst, Marc G. Vervloet

**Affiliations:** 1 Department of Nephrology, Amsterdam UMC, Amsterdam, The Netherlands; 2 Division of Nephrology, Department of Internal Medicine, University Medical Center Groningen, University of Groningen, Groningen, The Netherlands; 3 Department of Radiology & Nuclear Medicine, Amsterdam UMC, location VUmc, Amsterdam, The Netherlands; 4 Department of Epidemiology and Data Science, Amsterdam Public Health Institute, Amsterdam UMC, Amsterdam, The Netherlands

**Keywords:** Magnesium, Phosphate binder, Arterial stiffness, Chronic kidney disease, Calcification, Inflammation, Randomized controlled trial

## Abstract

**Background:**

Arterial stiffness and calcification propensity are associated with high cardiovascular risk and increased mortality in chronic kidney disease (CKD). Both magnesium and phosphate are recognized as modulators of vascular calcification and chronic inflammation, both features of CKD that contribute to arterial stiffness. In this paper, we outline the rationale and design of a randomized controlled trial (RCT) investigating whether 24 weeks of oral magnesium supplementation with or without additional phosphate-binding therapy can improve arterial stiffness and calcification propensity in patients with stage 3–4 CKD.

**Methods:**

In this multi-center, placebo-controlled RCT, a total of 180 participants with an estimated glomerular filtration rate of 15 to 50 ml/min/1.73 m^2^ without phosphate binder therapy will be recruited. During the 24 weeks intervention, participants will be randomized to one of four intervention groups to receive either magnesium citrate (350 mg elemental magnesium/day) or placebo, with or without the addition of the phosphate binder sucroferric oxyhydroxide (1000 mg/day). Primary outcome of the study is the change of arterial stiffness measured by the carotid-femoral pulse wave velocity over 24 weeks. Secondary outcomes include markers of calcification and inflammation, among others calcification propensity (T_50_) and high-sensitivity C-reactive protein. As explorative endpoints, repeated ^18^F-FDG and ^18^F-NaF PET-scans will be performed in a subset of participants (*n* = 40). Measurements of primary and secondary endpoints are performed at baseline, 12 and 24 weeks.

**Discussion:**

The combined intervention of magnesium citrate supplementation and phosphate-lowering therapy with sucroferric oxyhydroxide, in stage 3–4 CKD patients without overt hyperphosphatemia, aims to modulate the complex and deregulated mineral metabolism leading to vascular calcification and arterial stiffness and to establish to what extent this is mediated by T_50_ changes. The results of this combined intervention may contribute to future early interventions for CKD patients to reduce the risk of CVD and mortality.

**Trial registration:**

Netherlands Trial Register, NL8252 (registered December 2019), EU clinical Trial Register 2019-001306-23 (registered November 2019).

**Supplementary Information:**

The online version contains supplementary material available at 10.1186/s13063-022-06562-9.

## Administrative information

Note: the numbers in curly brackets in this protocol refer to SPIRIT checklist item numbers. The order of the items has been modified to group similar items (see http://www.equator-network.org/reporting-guidelines/spirit-2013-statement-defining-standard-protocol-items-for-clinical-trials/).

**Table:** 

Title {1}	Reversal Of Arterial Disease by modulating Magnesium and Phosphate (ROADMAP-study): rationale and design of a randomized controlled trial assessing the effects of magnesium citrate supplementation and phosphate binding therapy on arterial stiffness in chronic kidney disease
Trial registration {2a and 2b}.	Netherlands Trial Register, NL8252 (registered December 2019), EU clinical Trial Register 2019-001306-23 (registered November 2019). We affirm that all WHO Trial Registration Dataset items are included within the protocol and the majority of items are represented within these trial registries.
Protocol version {3}	15^th^ of April 2021 Version 3.3.
Funding {4}	The collaboration project is financed by the PPP Allowance made available by Top Sector Life Sciences & Health to The Dutch Kidney Foundation (16TKI02) under Health Holland grant no. LSHM17034-HSGF to stimulate public-private partnerships. Additional financial support from Fresenius Medical Care, NedMag and Calciscon AG
Author details {5a}	1. Department of Nephrology, Amsterdam UMC, Amsterdam, The Netherlands 2. Department of Internal Medicine, Division of Nephrology, University Medical Center Groningen, University of Groningen, Groningen, The Netherlands; 3. Department of Epidemiology and Data Science, Amsterdam Public Health Institute, Amsterdam UMC, Amsterdam, The Netherlands. 4. Department of Radiology & Nuclear Medicine, Amsterdam UMC, location VUmc, Amsterdam, The Netherlands.
Name and contact information for the trial sponsor {5b}	Prof. M.G. Vervloet, MD, PhD^1^ Department of Nephrology, Amsterdam UMC, location VUmc, Amsterdam, The Netherlands De Boelelaan 1117, 1081 HV Amsterdam, The Netherlands E-mail: m.vervloet@amsterdamumc.nl T: 020-4442673
Role of sponsor {5c}	As the sponsor the Amsterdam UMC, location VUmc ‘ Stichting VUmc’ is responsible for the study design, data collection, management and analysis and publication of the result. The funders have been involved in the study design through consortia meetings, and their role in other study processes and the study related activities have been pre-defined in a consortium agreement, which includes statements of unrestricted publication of study results regardless of results.

## Introduction

### Background and rationale {6a}

Cardiovascular disease (CVD) is the leading cause of death in patients with chronic kidney disease (CKD). Accumulating evidence demonstrates that progressive medial calcification of the arteria, aberrant mineral metabolism, and chronic low grade inflammation contribute to the disproportionally high risk of CVD and mortality in CKD [1–6]. Until recently, efforts to reduce this risk in CKD have mainly focused on targeting calcium and high phosphate levels. During the last years, also inhibitors of calcification, such as magnesium, gained interest.

Systematic reviews and meta-analyses of large observational studies in various populations have demonstrated an association between lower magnesium levels and an increased risk of CVD and mortality [7–9]. This association can be partially explained through the effect of magnesium on arterial stiffness. Arterial stiffness, acknowledged as a surrogate end point for CVD, can be assessed by pulse wave velocity (PWV) [10–13]. Observational studies in various stages of CKD, including end-stage renal disease (ESRD), have confirmed an independent association between PWV and fatal and non-fatal CVD and all-cause mortality [12–16]. Recently, two clinical trials in slightly obese participants demonstrated a clinically relevant reduction of PWV after 24 weeks of magnesium supplementation, most pronounced within the subgroup with higher arterial stiffness at baseline [17, 18]. Also, multiple in vitro and animal studies demonstrate that magnesium can effectively prevent mineralization in experimental models of vascular calcification and improve calcification propensity [19–22]. Clinically, quantifying calcification propensity has recently become feasible by measuring calciprotein particle (CPP) maturation time in vitro (T_50-_test) from patient serum samples, reflecting the transformation of soluble, fetuin-A bound, and calcium and phosphate containing primary CPPs (CCP1) towards more toxic, secondary CPPs (CPP2) [23, 24]. These CPP2 are alleged to cause vascular calcification, inflammation, and endothelial dysfunction, all features that affect arterial stiffness. In CKD, the T_50_ is lower compared to non-CKD individuals, and moreover, observational and clinical studies with CKD patients have linked a lower T_50_ and increased level of CPP2 to arterial stiffness, CVD, and mortality [24–31]. With increasing kidney impairment also derangements of phosphate homeostasis arise, with increasing fibroblast growth factor 23 (FGF23) preceding hyperphosphatemia, both associated with increased cardiovascular risk, arterial stiffness, and mortality [32–37]. Clinical trials in patients on hemodialysis have demonstrated that it is possible to improve T_50_ through lowering phosphate or increasing magnesium, respectively [38–40].

Currently, most magnesium intervention studies with focus on calcification propensity or arterial stiffness have been performed in a relatively healthy population or patients with ESRD [17, 40–42]. Clinical trials assessing the outcomes of phosphate-binding therapy in CKD patients with normophosphatemia are very limited [43–45]. Assuming that reducing phosphate levels inhibit rather than reverse established vascular calcification, patients with moderate CKD could benefit by preventing phosphate levels to increase. Furthermore, studies on the effect of magnesium supplementation or phosphate-binding therapy with the non-calcium containing phosphate binder sucroferric oxyhydroxide (SFOH) are not yet performed in stage 3 to 4 CKD patients with normophosphatemia. Combining magnesium supplementation and phosphate-lowering therapy could be a promising, early intervention in CKD patients aiming to reduce their severe cardiovascular risk.

In this paper, we will outline the design of a multi-center RCT investigating the effect of 24 weeks oral magnesium supplementation and additional phosphate-binding therapy, in patients with stage 3–4 CKD.

### Objectives {7}

The primary objective of this study is to assess the effect on arterial stiffness of the combined use of magnesium supplementation and phosphate binder therapy during 24 weeks in patients with stage 3–4 CKD. Secondary outcomes are several markers related to vascular calcification, such as calcification propensity, the amount of secondary CPPs formed, and markers of vascular inflammation and mineral bone metabolism, including FGF23, α-Klotho, intact parathyroid hormone (iPTH), and high-sensitivity C-reactive protein (hsCRP). In addition, explorative ^18^F-NaF and ^18^F-FDG positron emission tomography (PET) scans will be performed in a subgroup to evaluate the effects of the intervention on vascular calcification and inflammation, respectively.

### Trial design {8}

This study is a multi-center, randomized, placebo controlled, superiority trial. Stratified randomization allocates participants to four parallel intervention groups with a ratio of 1:1:1:1.

## Methods: participants, interventions, and outcomes

### Study setting {9}

Participants are included at four different study sites within the Netherlands: Amsterdam University Medical Centers (Amsterdam UMC) location VUmc and location AMC, University Medical Center Groningen (UMCG), and the peripheral hospital Onze Lieve Vrouwe Gasthuis (OLVG) location West, Amsterdam.

### Eligibility criteria {10}

#### Inclusion criteria

- Aged between 18-80 years and an estimated life expectancy of > 1 year
- CKD stages 3 and 4 (eGFR between 15 and 50 ml/min/1.73 m^2^)
- Plasma magnesium concentration 0.5*–*1.4 mmol/L
- Plasma phosphate concentration 0.8*–*1.6 mmol/L
- Written informed consent

#### Exclusion criteria

- Use of phosphate-binding therapy
- Renal transplantation in medical history or expected transplantation within 6 months
- Atrial fibrillation or atrial flutter at last clinical or screenings ECG
- Prolongation of QTc interval of > 500 ms, 2nd or 3rd degree atrioventricular block or bradycardia (heart rate below 50 bpm) on screenings ECG
- Known unstable carotid plaques
- Endoprosthesis of the aorta
- Hemochromatosis or other causes of iron overload, or hemoglobin > 10.5 mmol/L
- Chronic diarrhea or gastrointestinal absorption disorders
- Chronic use of antibiotics
- Active malignancy
- Pregnancy or lactation
- Serious substance abuse
- Recurrent incompliance for medication intake or hospital visits, i.e., “no-shows”
- Unwilling to discontinue over-the-counter magnesium supplements for the study duration
- Inability to measure PWV or to take blood samples for any reason, inability to swallow medication

#### Additional exclusion criteria for PET-scan eligibility

- Chronic inflammatory or auto-immune diseases
- Use of antibiotics or corticosteroids within the last 4 weeks
- Active infection (such as urinary tract infection, pneumonia, or wound infections) in 4 weeks prior to the PET scan
- Type 1 or 2 diabetes

### Who will take informed consent? {26a}

Written informed consent will be obtained and signed by the coordinating researcher (MD) or by a researcher/research nurse on their behalf before the start of any study related assessment.

#### Additional consent provisions for collection and use of participant data and biological specimens {26b}

Additional consent will be asked for the explorative PET-scans, as well as for data use and ‘biobank’ storage (including serum, plasma, whole blood and urine samples that are frozen at − 80°C for 15 years) for further research on vascular calcification kidney function.

### Interventions

#### Explanation for the choice of comparators {6b}

Magnesium citrate or placebo will be provided in a double blind manner. The placebo capsules are visually indistinguishable from the magnesium citrate capsules and contain Amylum Solanin (starch). An open-label design for the SFOH was chosen, because it was not feasible to create a convincing SFOH placebo (due to dark discoloration of stool and a distinguishing taste and color that could not be replicated or encapsulated without substantially increasing pill burden).

#### Intervention description {11a}

All participants will be randomly allocated to one of four interventions for a total of 24 weeks; see Table 1.

**Table 1:** Allocation groups

	***Allocation group***	***Active daily dose***	***Intake***
A	Magnesium citrate	350 mg^a^	3 dd 117mg
B	Placebo	x	3 dd 117mg
C	Magnesium citrate + SFOH	350 mg^a^, 1000 mg	3 dd 117mg + 2 dd 500mg^b^
D	Placebo + SFOH	x, 1000 mg	3 dd 117mg + 2 dd 500mg^b^

*SFOH* sucroferric oxyhydroxide, *dd* de die (per day)

^a^350 mg elemental magnesium, ^b^the SFOH intervention starts with one week of 1 dd 500 mg

Each allocation group receives one double blind intervention: magnesium citrate or matching placebo. Two allocation groups additionally receive the open label SFOH intervention.

The magnesium citrate and matching placebo capsules are prescribed thrice daily. Each magnesium citrate capsule contains 117 mg of elemental magnesium (magnesium citrate complex of 17.8%), resulting in a daily dose of 350 mg (14 mmol) magnesium. Magnesium citrate has a good bioavailability compared to other formulations and the laxative effect of magnesium citrate is limited [46]. To optimize absorption, the daily dose of 350 mg magnesium is distributed over the day, and this dosage is based on an intervention study that demonstrated a clinically relevant improvement of arterial stiffness after 24 weeks of supplementing 350mg magnesium citrate in slightly obese but healthy participants [17]. The magnesium citrate supplements and placebo are produced as one batch by Laboratory Medisan (Heerenveen, the Netherlands). Each package of magnesium citrate or placebo is labeled with a unique code that was randomly assigned by Laboratory Medisan accompanied with an identification list that was provided to the research pharmacies.

Participants that are randomized to arm C or D are requested to take the SFOH chewing tablet of 500 mg at the beginning of their two largest meals of the day. In order to limit potential gastro-intestinal side effects, participants start the first week of the intervention with SFOH 500 mg once a day whereafter they continue with twice daily 500mg for the remaining 23 weeks. A dose of 1000 mg/day is adequate in lowering phosphate levels in case of hyperphosphatemia [47] and could prevent phosphate from increasing over time without considerable risk of hypophosphatemia.

#### Criteria for discontinuing or modifying allocated interventions {11b}

**Fig. 1:**
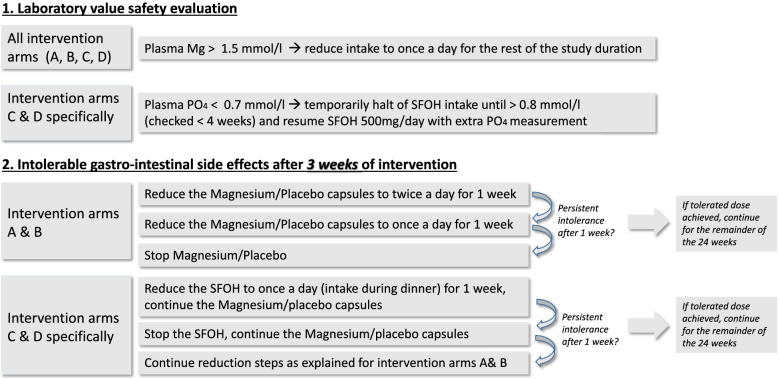
Criteria for discontinuation, medication modification, and follow-up. SFOH, sucroferric oxyhydroxide; Mg, magnesium; PO_4_, phosphate

Criteria for discontinuation or stepwise modification of the intervention in the context of safety evaluation and intolerable side effects, including subsequent follow-up, are summarized in Fig. 1.

#### Strategies to improve adherence to interventions {11c}

Medication intake, tolerability, and adherence is actively assessed during each study visit. Pill count will be performed at 12 and 24 weeks of intervention. Subjects will be considered compliant based on pill count when they take at least 80% of the study medication. In addition to plasma levels of magnesium and phosphate, also 24-h urinary magnesium and phosphate concentrations at baseline and after 24 weeks of intervention will be supportive in the evaluation of adherence.

#### Relevant concomitant care permitted or prohibited during the trial {11d}

Magnesium supplements or magnesium containing antacids and laxatives are discontinued after informed consent ensuring a wash-out period of at least 4 weeks before the start of the intervention (baseline). A magnesium-free alternative is considered when necessary. The use of phosphate binders is not allowed with the exception of calcium carbonate. Contrary to its phosphate-lowering effects in a hemodialysis population, calcium carbonate does not have a relevant nor significant serum phosphate-lowering effect in patients with stage 3–4 CKD [48]. Concomitant phosphate binder therapy is allowed in case of rising phosphate levels with the need of medical intervention conform KDIGO guidelines and decided by the treating physician. This will be registered during the trial and these subjects will maintain in the trial based on intention-to-treat (ITT) analysis. In case of phosphate levels of ≥ 1.60 mmol/l, the treating physician will actively be notified by the research team. Physicians are stimulated to treat all participants according to current standard of care including aiming for appropriate blood pressure, the preferential use of inhibitors of RAS, and the use of statin therapy according to KDIGO guidelines.

Although a stable diet is encouraged for the entire study period, dietary changes following physician or dieticians’ advice are allowed. Therefore, dietary changes will be recorded retrospectively at the end of intervention period.

#### Provisions for post-trial care {30}

There is no need and therefore no provisions for ancillary or post-trials care were made. However, the sponsor has a liability insurance which is in accordance with article 7 of the WMO. This insurance provides cover for damage to research subjects through injury or death caused by the study. The insurance applies to the damage that becomes apparent during the study or within 4 years after the end of the study.

#### Outcomes {12}

The primary endpoint of the study is difference in change of PWV over 24 weeks between the active intervention groups compared to the placebo group. PWV is measured at baseline and 12 and 24 weeks, with the SphygmoCor v9 (AtCor Medical, Sydney Australia). Before measurement of the PWV, blood pressure measurements in supine position are repeatedly performed until the blood pressure stabilizes with a maximum systolic difference of ≤ 3mmHg. The carotid-femoral PWV is measured at least twice during these corresponding visits, with a tonometer and simultaneous ECG recording. The carotid-femoral transit time is automatically calculated based on the delay of the pulse wave arrival at carotid and femoral measurement sites in relation to the ECG R-wave. Subsequently, the PWV is calculated using this carotid-femoral transit time and the travel distance between the carotid artery and femoral artery measurement sites. The travel distance is assessed as the direct carotid-femoral distance (in mm) * 0.8, as is recommended in a PWV expert consensus document [49]. Each measurement is evaluated based on strict quality criteria and the average of two good quality measurements is taken (see Additional file 1).

Secondary endpoints include the difference in calcification propensity (T_50-_test), (secondary) CPP concentrations and the difference in serum/plasma concentrations of α-Klotho, C-terminal (c)FGF23, hsCRP, phosphate, and magnesium, over 24 weeks between groups. All measurements are performed in samples collected after at least 3 h of fasting. Serum for the T_50-_test and the gel filtration method based quantification of CPP measurements is frozen at − 80 °C and will be measured as described previously [24, 50]. The T_50-_test established inter-assay coefficients of variation of standards precipitating at 120, 260, and 390 min are 7.8%, 5.1%, and 5.9%, respectively [24]. These measurements will be performed as one batch without additional freeze-thaw. EDTA-plasma concentrations of cFGF23 and iPTH, and serum α-Klotho are centrally measured at the Amsterdam UMC, after storage at -80 °C at participating sites. Routine blood tests are performed directly after withdraw at the laboratories of participating centers, including plasma magnesium, phosphate, creatinine, hsCRP, calcium, albumin, potassium, hemoglobin, ferritin, lipid spectrum (low density lipoprotein (LDL), high density lipoprotein (HDL), total cholesterol and triglycerides), fasting glucose, and bicarbonate. The eGFR is calculated using the creatinine based CKD-EPI (Chronic Kidney Disease Epidemiology Collaboration) equation and plasma calcium is corrected for albumin concentration [calcium _mmol/L_ + 0.016 × (40 – albumin _g/L_)]. In addition, urinary magnesium, phosphate, creatinine, sodium, and protein are determined in 24-h urine samples at baseline and at 24 weeks of intervention. Additional parameters include demographic characteristics, anthropometric measurements, and medical history at baseline. Also seated blood pressure measurements are performed at baseline and 12 and 24 weeks with an oscillometric, automatic blood pressure monitor averaging a second and third measurement. An overview of all study measurements and procedures are presented in Table 2.

**Table 2:**
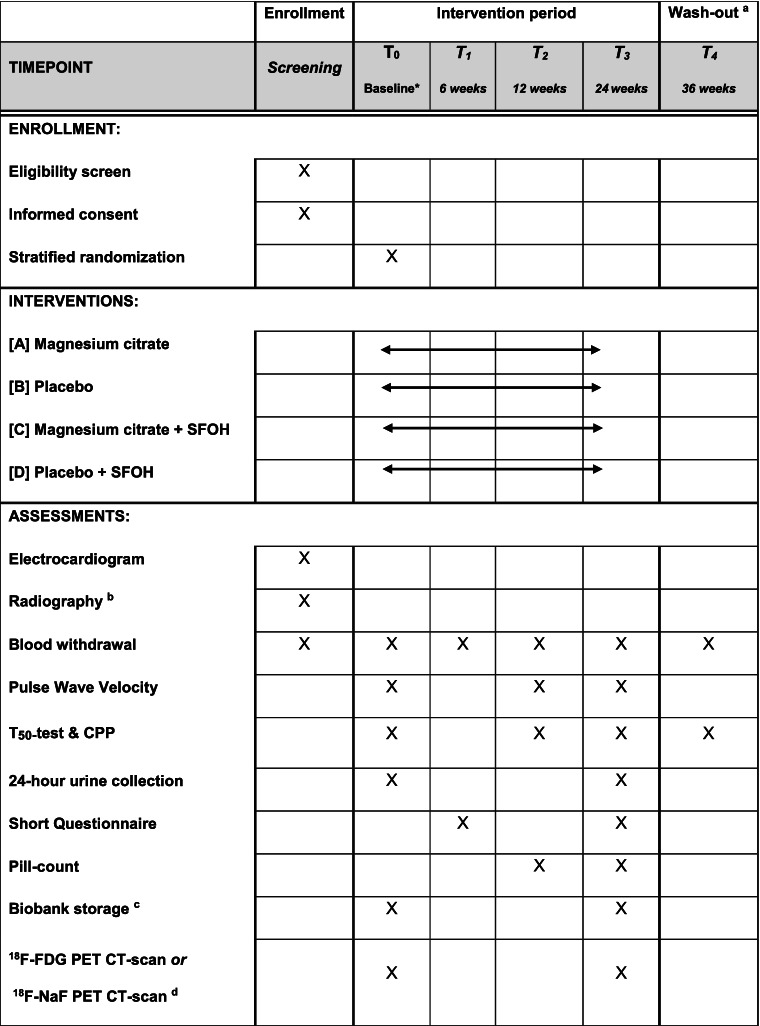
Timeline of study visits, intervention, and assessments of the ROADMAP-study

*Abbreviations*: *SFOH* sucroferric oxyhydroxide, *h* hour, ^*18*^*F-FDG* fluor-18-deoxyglucosev, ^*18*^*F-NaF* fluor-18-sodium fluoride, *PET* positron emission tomography, *CT* computer tomography

^*^Baseline follows at least 4 weeks after screening with a maximum of 3 or 6 months for participants with an eGFR of < 30 or ≥ 30 ml/min/1.73m^2^ respectively

^a^The wash-out visit is an optional visit

^b^X-ray of the lower vertebra (X-LWK) in order to score calcification with the Kauppila index

^c^Including serum, plasma, and 24 h urine samples, only stored for participants with additional Biobank informed consent

^d^Subsample of 40 participants with additional PET-scan consent, resulting in 10 participants with ^18^F-FDG or ^18^F-NaF PET-scan in each intervention arm

#### Participant timeline {13}

Table 2 presents a time line including all study visits, interventions and assessments for the entire study period. In order to combine study visits with regular hospital or laboratory visits, the planning of the study visits are flexible with a ± 7 day margin. The baseline visit follows at least 4 weeks after screening (potential magnesium wash-out period) with a maximum of 3 or 6 months for participants with an eGFR of < 30 or ≥ 30 ml/min/1.73m^2^, respectively.

#### Sample size {14}

The sample size of this study is based on the primary endpoint. A 1.0 m/s difference in PWV is clinically relevant and can be expected with a magnesium intervention of 24 weeks in healthy subjects and a CKD population with accelerated CVD risk [11, 17]. Based on prior studies on arterial stiffness in populations with CKD stages 1–5, with mean PWV values between 10.5 and 12.0 m/s and a standard deviation (SD) of 1.4-3.5 m/s [10, 12, 13], we assume for our study population with an eGFR 15–50 ml/min/1.73m^2^ a SD of 2.0 m/s. With a statistical power of 80%, a two-sided significance of 0.05 and a rho of 0.7 to account for the correlation between baseline and follow-up measurements, 38 participants are required per group. To account for an anticipated drop-out rate of 15% during the 24 weeks, a total of 180 CKD patients will be included and randomized in a ratio of 1:1:1:1, with 45 participants in each intervention group. In case of an unexpected high drop-out rate (> 15%) for reasons not related to study participation (for example starting dialysis, transplantation, referral to a non-participating center), then additional participants can be recruited.

The sample size calculation was performed with the PASS 15.0.5 software and a statistical consultant.

#### Recruitment {15}

Eligible subjects will be informed about the study by their treating physician in the outpatient clinic setting of all participating centers. Those patients who show interest and consent will receive written information and a phone call by one of the research team members to provide more information and handle questions. To reduce potential participation barriers, participants can consent to full study participation, or they can choose to participate without the additional randomization for PET scans, without biobank storage and/or skip the last washout visit.

### Assignment of interventions: allocation

#### Sequence generation {16a}

The randomization procedure is performed by the research pharmacy with ALEA software (version 16) with stratification for the eGFR at screening of < or ≥ 30ml/min/1.73m2 and a Kauppila index of < or ≥5. The Kauppila index is determined at the run-in visit by a lateral lumbar radiography depicting presence of calcification in the abdominal aorta [51].

#### Concealment mechanism {16b}

None of the research team members have access to the ALEA randomization environment. Only the research pharmacy responsible for stratified randomization and the independent research nurse responsible for randomization of the PET-scans have individual passwords to access the randomization results. The identification list of unique randomized numbers on the medication bottles is only provided to the pharmacies. All assessments, including PWV measurement and laboratory assessments, are performed without knowledge of the allocation. Statistical analyses will be performed based on an intervention number provided by the pharmacy and only afterwards the corresponding intervention will be revealed.

#### Implementation {16c}

After initial approach by their treating physician, participants will be enrolled by one of the researchers. After informed consent and screening, participants are allocated by the research pharmacies through ALEA software. A subsample of the first 40 participants who met additional inclusion criteria and gave their consent for nuclear imaging will be randomized to undergo either explorative ^18^F-FDG or ^18^F-NaF PET-scan imaging at the VUmc. To ensure five participants of each nuclear tracer in all four intervention groups, this randomization is performed manually (i.e. an envelope for each allocation arm, including 5 PET-scan tickets for each tracer) by an independent research nurse with access to the ALEA randomization results.

### Assignment of interventions: blinding

#### Who will be blinded {17a}

The randomized, double-blind design ensures that participants as well as the research team and care providers will be blinded for the magnesium citrate intervention. The magnesium citrate and placebo identification list will only be available to concerning research pharmacies. Randomization for the additional SFOH prescription is open-label and therefore known to all involved. Data analyses will be performed before revealing the randomization code.

#### Procedure for unblinding if needed {17b}

Only in case of a suspected unexpected serious adverse reaction (SUSAR) the pharmacist will be notified by the coordinating researcher to disclose the intervention allocation of the concerning participant to the independent expert. If the independent expert develops concerns, these will be discussed with the research team and the METC if applicable.

### Data collection and management

#### Plans for assessment and collection of outcomes {18a}

Data collection of the primary outcome will be performed in duplicate, striving for an average PWV based on two good quality measurements during the concerning visits. Only three trained research team members will perform the PWV measurements and pre-specified quality criteria of the PWV assessment are summarized in Additional file 1. To prevent inter-assay variation laboratory measurements of T_50,_ CPPs, iPTH, cFGF-23, and Klotho will be measured centrally in one or limited batches. Since it is known freeze thaw cycles influence T_50-_test and CPP measurement, samples will be frozen directly at − 80 °C and only thawed once for central measurement.

Radiologic assessment for the Kauppila index in the context of stratification is performed by the coordinating researcher, with prior established good interrater agreement (kappa of 0.89) in a test set that was scored by the trained researcher and an experienced radiologist. All PET-scans will be performed at the Philips Vereos PET CT-scanner of the Amsterdam UMC, location VUmc. Assessment of the nuclear images of ^18^F-NaF and ^18^F-FDG PET-scans will be performed by a trained researcher after finalization of all scans to reduce intra-rater reliability.

#### Plans to promote participant retention and complete follow-up {18b}

To promote participant retention and complete follow-up, contact through phone and email is encouraged and planning of visits are confirmed through email. Also, great effort is made to combine regular laboratory or physician hospital visits with research measurements to facilitate research investment. Participants who discontinue participation after the start of the intervention will be asked to accept the T3 PWV measurement to allow ITT analysis of the primary endpoint. Protocol deviations regarding the intervention will be handled in the analyses differentiating between ITT and on treatment analyses.

#### Data management {19}

Castor electronic data capture serves as a clinical data management system, capturing all study data in electronic case report forms (eCRF) conform local METC requirements. To ensure correct data entry, embedded data validation and dependencies are applied and warnings appear in case of data entry outside the reference ranges. Source documents are stored in the trial master file (TMF), investigator site file (ISF) or electronic patient dossier (EPD).

#### Confidentiality {27}

Potential participants are pre-screened through an automatic report function, selecting patients based on pre-specified criteria. Selected participants are approached by their treating physician and only after consent of the patient full access to their EPD is allowed, with subsequent first contact by a research team member. After informed consent, each participant of the study will receive a not reducible, unique study code, which will be used for all study related documents and the eCRF. Except for age, sex, and ethnic group, no other identifying data will be used. The participant identification log is stored separately and only accessible to the coordinating researchers at the concerning study sites and the responsible monitor.

#### Plans for collection, laboratory evaluation, and storage of biological specimens for genetic or molecular analysis in this trial/future use {33}

In case of “biobank” consent additional serum, plasma, whole blood, and 24-h urine samples are collected at baseline and after 24 weeks intervention. This material is frozen at − 80°C and stored for 15 years for future research regarding vascular calcification, inflammation, endothelial dysfunction, and genetics in CKD.

### Statistical methods

#### Statistical methods for primary and secondary outcomes {20a}

Patient characteristics will be presented by treatment arm as mean ± standard deviation (SD) or median with an interquartile range (IQR) for normally and non-normally distributed continuous data, respectively. Categorical variables will be presented as number and percentage. Differences between treatment arms will be visually inspected for clinically relevant differences.

The primary and secondary endpoints will be analyzed with linear mixed models, with repeated measurements (at baseline, 12 weeks, and 24 weeks) and random intercepts at patient level. In case of non-normally distributed residuals of the outcome variables, suitable transformation (e.g., logarithmic) will be performed. Based on the −2 log likelihood evaluation, additional center level and random slopes will be added to the analysis when needed. The main analysis will focus on the three active intervention groups separately, in comparison to the placebo. Also, the additional value of the combined intervention of magnesium citrate and SFOH will be analyzed compared to solely magnesium citrate and SFOH supplementation by coding the magnesium citrate and SFOH as reference category, respectively. Due to stratified randomization, these important confounders are expected to be evenly distributed between the intervention groups. However, confounding by baseline differences of age, sex, eGFR, Kauppila index, baseline plasma magnesium, and phosphate concentrations will be considered in case of clinically relevant or ≥ 10% difference between groups. To evaluate when the effect of the intervention occurs at its maximum, an interaction term for both categorical time variables (12 and 24 weeks versus baseline) will be evaluated in the analysis for primary and secondary outcomes.

The primary and secondary outcomes will be investigated as an ITT analysis. Within the ITT analyses, all participants will be included who have been randomized and participated in the baseline measurements and at least one follow-up visit. This analyses will be based on the initial treatment assignment and not on the treatment eventually received, thus not taking in to account dose alterations or cessation of (part of) the study intervention. Results will be considered statistically significant with a *P*<0.05 (2-sided) for the primary endpoint. For secondary endpoint analyses, we will mainly focus on effect estimates and confidence intervals. Data analysis will be performed in the SPSS 24.0 software for Windows (SPSS incorporated, Chicago, Il, USA) and StataSE 14 (StataCorp, College Station, USA) and will be performed blinded to treatment allocation.

#### Interim analyses {21b}

No interim analyses will be performed. However, halfway study recruitment a recalculation of the sample size will be performed based on the PWV SD, rho, and percentage of dropout until then, with adjustment of the sample size if applicable.

#### Methods for additional analyses (e.g., subgroup analyses) {20b}

The intervention effect on plasma magnesium and phosphate and their urinary excretion will be analyzed with linear mixed models with repeated measurements and linear regression analysis, respectively. Subgroup sensitivity analyses will be reported for the stratified eGFR, Kauppila index, sex, median age, blood pressure, presence of diabetes, median baseline magnesium, and phosphate levels and proton pump inhibitor or diuretic use to test the robustness of the analysis. In addition, for the primary endpoint of PWV, the mediating effect by plasma magnesium and phosphate levels, blood pressure, T_50-_test, CPPs, α-klotho, and cFGF-23 will be explored using causal mediation analysis.

The analysis of the explorative ^18^F-NaF and ^18^F-FDG PET-scans will be based on the axial regions of interest (ROI). Differences between the intervention groups in standardized uptake value (SUV), target to background ratio (TBR), and most diseased segment (MDS) will be analyzed with linear regression analysis, with adjustment for baseline value.

#### Methods in analysis to handle protocol non-adherence and any statistical methods to handle missing data {20c}

To evaluate the influence of non-adherence (treatment adherence < 80% based on pill-count), an on-treatment analysis will be performed in addition to the ITT analyses. Missing outcome variables will not be imputed since mixed models can handle these missing data. In case of missing data in relevant covariates, multiple imputation with predictive mean matching (PMM) will be performed.

#### Plans to give access to the full protocol, participant level-data, and statistical code {31c}

Access to the full study protocol and statistical scripts can be requested after study finalization and publication. Coded participant level data will only be shared in case of consent and compliance with the established consortium agreement.

### Oversight and monitoring

#### Composition of the coordinating center and trial steering committee {5d}

Professor M.G. Vervloet is the primary investigator (PI) of the Amsterdam UMC, location VUmc and location AMC. Coordinating center is the Amsterdam UMC, location VUmc. Participating study sites include the UMCG (PI Professor. M.H. de Borst) and the affiliated hospital OLVG (PI Dr. C.E.H. Siegert). The coordinating researcher (E.A. Vermeulen, MD) and research nurse (M. van de Putte) are both providing the day to day support of the study at both locations of the Amsterdam UMC and the OVLG. Day to day support and study measurements at location UMCG are the responsibility of Dr. C. Eelderink.

This trial is embedded within the NIGRAM2+ consortium that has regular meetings every few months including progress meetings and reports to the Dutch Kidney foundation and Health Holland.

#### Composition of the data monitoring committee, its role and reporting structure {21a}

A data safety monitoring board or safety committee is not established for this study since this is a low risk study. On site monitoring of all study sites is performed regularly by an independent monitor of the Clinical Research Bureau (CRB) of the Amsterdam UMC, location VUmc as explained in more detail in the section “Frequency and plans for auditing trial conduct (Spirit Guidance {23})”. For more information, visit https://www.amsterdamumc.org/en/research/support/about/clinical-research-monitoring.htm.

#### Adverse event reporting and harms {22}

The following, prespecified AEs, reported spontaneously by the subject or observed by the investigator or research staff will be recorded within the electronic case report forms (eCRF): gastro-intestinal complaints, muscle weakness, arrhythmias, fractures, somnolence, dizziness, tooth discoloration, disturbance of taste, plasma magnesium <0.5 or >1.5 mmol/L or plasma phosphate <0.7 or >1.6 mmol/L. A short questionnaire is administered at 6 and 24 weeks of intervention, evaluating self-reported side effects and convenience, based on two domains of the Treatment Satisfaction Questionnaire for Medication-14 (TSQM-14); see Additional file 2 [52]. AEs will be presented as descriptive data in a table split by allocation arm.

#### Frequency and plans for auditing trial conduct {23}

All study sites will be monitored by an independent monitor of the CRB of the Amsterdam UMC, location VUmc. In addition to a preparatory and closing visit, each study site will have monitoring visits every 6 to 12 months. The monitors will review the source documents to determine whether the data reported in the eCRF are complete and accurate. Also correct use of inclusion and exclusion criteria, data protection as well as medical research products will be verified.

#### Plans for communicating important protocol amendments to relevant parties (e.g., trial participants, ethical committees) {25}

In case of any substantial change in the protocol, an amendment will be submitted to the METC and the CA and the research team and participants will be notified. If applicable, also the participant information letter, the informed consent form, study operator manuals, and trial registrations will be updated. After the start of the study, one substantial amendment has been submitted. The current study protocol (version 3.3, 15 April 2021) is approved on 28 April 2021. Changes in the original approved protocol are summarized in Additional file 3.

#### Dissemination plans {31a}

All results will be reported in medical journals with immediate open access conform the NIGRAM2+ consortium agreement, regardless of results. When granted the opportunity, the results will be presented at national and international conferences. Main study results will be communicated actively to all health care professionals and participants that participated in the study. Also the Dutch organization for Kidney patients “Nierpatiënten Vereniging Nederland” will be approached for dissemination to their members, and we aim to highlight our results through https://www.niernieuws.nl/ and similar kidney patient platforms.

## Discussion

This study is the first multi-center, randomized controlled trial assessing the combined effect of magnesium citrate and the phosphate binder SFOH on arterial stiffness, calcification, and inflammation parameters in people with stage 3 and 4 CKD, based on the understanding that increasing magnesium and improving phosphate homeostasis could synergistically retard the progression of established vascular calcification [19], and people with moderate CKD could benefit most. To our knowledge, only two oral magnesium intervention studies in patients with moderate CKD have been performed so far [53, 54]. A 2-year open-label randomized trial in stage 3–4 CKD patients demonstrated a statistically significant smaller median increase in coronary artery calcification (11.3% versus 39.5%) for participants treated with magnesium oxide as compared to the control group [53]. Another RCT in a comparable population with moderate CKD resulted in a significant increase (improvement) of T_50_ of 40 min (95% confidence interval 11–70 min, *P* <0.05) compared to placebo, after 8 weeks of oral magnesium hydroxide supplementation [54]. Different form the established effects of phosphate binders on correcting hyperphosphatemia, these effects of phosphate-lowering therapy in moderate CKD in the absence of hyperphosphatemia are not yet well established [43–45, 55, 56]. In this study, the non-calcium based phosphate binder SFOH is prescribed because of its relatively low pill burden compared to other phosphate binders [47] and to avert the potential confounding effect on vascular calcification due to calcium content. The explorative ^18^F-FDG and ^18^F-NaF PET-scans will also contribute to the understanding of important pathophysiological mechanisms of arterial wall inflammation and calcification, respectively. A recent study in CKD patients without overt atherosclerotic disease observed increased arterial wall inflammation through ^18^F-FDG PET imaging, compared to healthy controls. ^18^F-NaF PET-scans conceivably enable microcalcification formation detection, even before macroscopic calcification is revealed through regular CT techniques. Moreover, the lack of ^18^F-NaF uptake in macroscopic calcified vessel walls supports the theory that ^18^F-NaF only binds to active calcification deposition and therefore studies an early or active phase of arterial calcification. It is in this early phase, that modulating magnesium and phosphate might prove most beneficial.

Several clinical and preclinical studies support the potential of combined magnesium and phosphate interventions. In vitro, the presence of high magnesium levels can prevent the phosphate-induced phenotypic switch of vascular smooth muscle cells of the intima media into bone forming cells and additionally is an inhibitor of the formation of precipitating calcium-phosphate products (hydroxyapatite nanocrystals) [21, 57, 58]. In clinical setting, high magnesium concentrations are associated with lower calcification propensity (i.e., higher T_50_ values), inhibition of the phosphate containing CPP formation rate, and with lower FGF23 levels [54, 59].

Strengths of this study include the possibility to investigate magnesium citrate and sucroferric oxyhydroxide as modulators of vascular calcification synergically and separately on various parameters of interest. By additionally measuring the T_50_, we will be able to investigate if the presumed effect on arterial stiffness is mediated by calcification propensity. The study design with repeated measurements of primary and secondary outcomes allows for thorough statistical analyses and a reduced number of participants. This design also allows for studying the intervention effects over time, i.e., at 12 weeks, 24 weeks, and after a wash-out period. The main limitation of this study is the fact that arterial stiffness is only a intermediate marker of CVD and has some degree of operator dependency. However, the association of PWV and cardiovascular disease and mortality is well established in the CKD population [11–14], and intervention effects can be detected already after 6 months [17, 18]. The PWV measurements will be performed by a limited number of well-trained study personnel, aiming at measurements performed on an individual participant by the same operator, thereby limiting the effect of inter-operator variability.

A limitation of the study design is the open-label phosphate binder intervention with SFOH chewing tablets. A double-blind design with a SFOH placebo was not feasible, due to the notable structure, color, and taste of these tablets and the frequently encountered side effect of stools discoloration. However, the primary and secondary outcomes are standardized measurements and therefore unlikely to be influenced by this knowledge. Another limitation of this study is the fact that CKD patients frequently receive dietary consultation, stimulating a more healthy diet and specifically targeting potassium and phosphate intake. Therefore, subsequent dietary changes during the follow-up period could influence magnesium and phosphate levels. However, good quality dietary records are notoriously difficult and therefore we did not consider this in our analysis.

This RCT will add knowledge on calcium-free phosphate binder therapy in normophosphatic patients with stage 3–4 CKD, and it investigates the effects of a magnesium supplement as potential intervention to improve vascular function. By doing so, this study aims to provide a rationale to early treatment for people with moderate CKD to reduce the high burden of CVD in this population.

## Trial status

Current trial status is ongoing. The study started 15 July 2020 with temporarily halt of recruitment due to COVID-19-related restrictions. Recruitment is expected to be completed in 2023 if COVID-19-related restrictions do not further hamper study recruitment.

## Supplementary Information

**Additional file 1.** Standard Operating Procedure of the Pulse Wave Velocity measurement and quality criteria.

**Additional file 2.** Questionnaire.

**Additional file 3.** Protocol changes compared the original approved protocol.

## Data Availability

The NIGRAM2+ consortium agreement is leading regarding data availability and publication.

## Abbreviations

AE: Adverse event
AMC: Academic Medical Center (together with VUmc ‘Amsterdam UMC’)
AVG: Personal Data Protection Act. Dutch translation: Algemene verordening gegevensbescherming, formerly mentioned as Wet bescherming persoonsgegevens (Wpb)
BMI: Body mass index
CA: Competent authority
CCMO: Central Committee on Research Involving Human Subjects; in Dutch: Centrale Commissie Mensgebonden Onderzoek
CKD: Chronic kidney disease
CPP: Calciprotein PARTICLES
CT: Computer TOMOGRAPHY
CVD: Cardiovascular disease
DSMB: Data Safety Monitoring Board
eCRF: Electronic case report form
ECG: Electrocardiography
eGFR: Estimated glomerular filtration rate
EPD: Electronic patient dossier
ESRD: End-stage renal disease
EudraCT: European Drug Regulatory Affairs Clinical Trials
^18^F-FDG: Fluor-18-deoxyglucose
^18^F-NaF: Fluor-18-sodium fluoride
FGF23: Fibroblast growth factor 23
KDIGO: Kidney Disease Improving Global Outcomes; global nonprofit organization developing and implementing evidence-based clinical practice guidelines in kidney disease
hsCRP: High-sensitivity C-reactive protein
ISF: Investigator site file
METC: Medical research ethics committee (MREC); in Dutch: medisch ethische toetsing commissie
mmol/L: Millimole/liter (0.001 mol per liter)
MDS: Most diseased segment
NIGRAM2+: Nijmegen Groningen Amsterdam consortium and acronym for ‘Nier Gericht Research van Arterie tot Mens’ with the 2+ referring to the follow-up of the first NIGRAM consortium, and the focus on the Mg^2+^ ion
OLVG: Onze Lieve Vrouwe Gasthuis
PET: Positron emission tomography
PMM: Predictive mean matching
RAS: Renin angiotensin system
ROI: Region of interest
iPTH: Intact parathyroid hormone
PWV: Pulse wave velocity
SAE: Serious adverse event
SD: Standard deviation
SFOH: Sucroferric oxyhydroxide
Sponsor: The sponsor is the party that commissions the organization or performance of the research, for example a pharmaceutical company, academic hospital, scientific organization or investigator. A party that provides funding for a study but does not commission it is not regarded as the sponsor, but referred to as a subsidizing party
SUSAR: Suspected unexpected serious adverse reaction
SUV: Standardized uptake value
T_50_: Calcification propensity (also referred to as CPP maturation time or calcification blood test)
TBR: Target to background ratio
TMF: Trial master file
UMC: University Medical Center(s)
UMCG: University Medical Center Groningen
VSMC: Vascular smooth muscle cell
VUmc: VU medical center (in Dutch: Vrije Universiteit medisch centrum)
WMO: Medical Research Involving Human Subjects Act (in Dutch: Wet Medisch-wetenschappelijk Onderzoek met Mensen
X-LWK: X-Lumbale Wervel Kolom, an X-ray of the lower back displaying the abdominal aorta
