# Supplementary material for: Reversal Of Arterial Disease by modulating Magnesium and Phosphate (ROADMAP-study): rationale and design of a randomized controlled trial assessing the effects of magnesium citrate supplementation and phosphate-binding therapy on arterial stiffness in moderate chronic kidney disease
Source: Trials. 2022 Sep 12;23:769. doi: 10.1186/s13063-022-06562-9 (PMC9465140; doi:10.1186/s13063-022-06562-9)
Supplement: Supplementary file 1 — Additional file 1. Standard Operating Procedure of the Pulse Wave Velocity measurement and quality criteria. [file 13063_2022_6562_MOESM1_ESM.pdf]

Additional file 1. Standard Operating Procedure of the Pulse Wave Velocity measurement and quality criteria.

Instructions prior to the visit

- Fast for at least 3 hours prior to the measurement
- Abstain from alcohol and caffeine at least 3 hours prior to the measurement
- Rest (supine position) for at least 5 minutes prior to the measurement

Preparation and positioning

- The measurement is performed in a quiet room without any disturbances
- Room temperature (approximately 20 - 22 °C)
- The subject is placed in fully supine position without cushion and facing strait upwards
- The three electrocardiogram electrodes connected and groin exposed
- Repeated blood pressure measurements until stabilization (defined as systolic and diastolic difference of ≤ 3mmHg)
- Marking of punctum maximum for carotid and femoral artery pulsation
- Measurement of direct carotid-femoral distance (used for calculation of PWV, see last header) and distance between carotid artery to suprasternal notch and belly button to femoral artery (for refining punctum maximum next measurement).

Data entry before measurement

- Study number and year of birth
- Initials of the operator
- Last systolic and diastolic blood pressure (after stabilization as defined above)
- Direct carotid-femoral distance (not yet corrected to avoid correction errors during measurement)

PWV measurement

- Measurement of the carotid and subsequent femoral pulse wave with the tonometer (placed on the predefined punctum maximum)
- Repeating measurement until two good quality PWV measurements if possible. In case of best measurement outside the punctum maximum marking as determent in the preparation step, new marking and corrected measurements are performed and used for next measurement.

Quality criteria PWV measurement

- Heart rate difference between carotid and femoral measurement ≤ 5 bpm
- The R-wave is clearly defined and has the highest amplitude of the ECG signal
- The foot of the pressure waveform (i.e. the initial upstroke) is clearly identified
- Stable and good quality pressure waveforms
- SD values of the “ECG-CAR” and “ECG-FEM” are acceptable /depicted in green (i.e. ≤ 6%)

Calculation of PWV during each visit

- Calculating the distance corrected PWV (m/s) by multiplying the PWV by 0.8, based on the formula of common carotid artery to common femoral artery distance *0.8 advised by the expert consensus document on PWV measurement (49).
- Calculating the mean of two (distance corrected) good quality PWV measurements per visit, if the difference between the measurments is less than 0.5 m/s. If the difference between the two measurements is > 0.5 m/s a third third measurement is taken if possible and the median value will be leading.
- In case of only one good quality measurement this single (distance corrected) PWV will be leading, disregarding those not meeting quality criteria. In case none of the measurements meet the quality criteria, the PWV will be reported as missing due measurement error.
