# Supplementary material for: Reversal Of Arterial Disease by modulating Magnesium and Phosphate (ROADMAP-study): rationale and design of a randomized controlled trial assessing the effects of magnesium citrate supplementation and phosphate-binding therapy on arterial stiffness in moderate chronic kidney disease
Source: Trials. 2022 Sep 12;23:769. doi: 10.1186/s13063-022-06562-9 (PMC9465140; doi:10.1186/s13063-022-06562-9)
Supplement: Supplementary file 2 — Additional file 2. Questionnaire. [file 13063_2022_6562_MOESM2_ESM.pdf]

**Additional file 2.** Questionnaire

# Questionnaire ROADMAP-study

*Study number.* _ _ _ _ _ *Moment:* *T _ _* *Date: _ _ /_ _/_ _ _*

**1. As a result of taking this medication, do you experience any side effects at all?**

*Yes* ☐ *No* ☐ → ***If 'No' you may skip question 2 to 5. Please continue with question 6***

**2. What side effects do you experience?**

. . . . . . . . . . . . . . . . . . . . . . . . . . . . . . . . . . . . . . . . . . . . . . . . . . . . . . . . . . . . . . . .

. . . . . . . . . . . . . . . . . . . . . . . . . . . . . . . . . . . . . . . . . . . . . . . . . . . . . . . . . . . . . . . .

**3. How bothersome are the side effects of the medication you take to treat your condition?**

*Not at All* — *Extremely bothersome*

☐ ☐ ☐ ☐ ☐

**4. To what extent do the side effects interfere with your physical health and ability to function (i.e., strength, energy levels etc.)?**

*Not at All* — *A Great Deal*

☐ ☐ ☐ ☐ ☐

**5. To what extent do the side effects interfere with your mental function (i.e., ability to think clearly, stay awake etc.)?**

*Not at All* — *A Great Deal*

☐ ☐ ☐ ☐ ☐

**6. How easy or difficult is it to use the medication in its current form?**

*Very easy* — *Extremely Difficult*

**7. How easy or difficult is it to plan when you will use the medication each time?**

| *Very easy* | | | | *Extremely difficult* |
|---|---|---|---|---|
| ☐ | ☐ | ☐ | ☐ | ☐ |

**8. How convenient or inconvenient is it to take the medication as instructed?**

| *Very convenient* | | | | *Extremely inconvenient* |
|---|---|---|---|---|
| ☐ | ☐ | ☐ | ☐ | ☐ |

**9. Did your study medication change compared to previous study visit?**

| *Yes* | *No* | |
|---|---|---|
| ☐ | ☐ | → ***If yes, please specify:*** |

Which medicine? . . . . . . . . . . . . . . . . . . . . . . . . . . . . . . . . . . . . . . . . . . . . . . . . . . .

By whom?: . . . . . . . . . . . . . . . . . . . . . . . . . . . . . . . . . . . . . . . . . . . . . . . . . . . . . . . .

When? . . . . . . . . . . . . . . . . . . . . . . . . . . . . . . . . . . . . . . . . . . . . . . . . . . . . . . . . . .

Why? : . . . . . . . . . . . . . . . . . . . . . . . . . . . . . . . . . . . . . . . . . . . . . . . . . . . . . . . . .

**10. Do you sometimes skip medication?**

| Frequently *(daily)* | Regularly *(few times a week)* | Sometimes *(Once a week)* | Rarely | Never |
|---|---|---|---|---|
| ☐ | ☐ | ☐ | ☐ | ☐ |

*This is the end of this questionnaire. Thank you!*
