# Supplementary material for: Reversal Of Arterial Disease by modulating Magnesium and Phosphate (ROADMAP-study): rationale and design of a randomized controlled trial assessing the effects of magnesium citrate supplementation and phosphate-binding therapy on arterial stiffness in moderate chronic kidney disease
Source: Trials. 2022 Sep 12;23:769. doi: 10.1186/s13063-022-06562-9 (PMC9465140; doi:10.1186/s13063-022-06562-9)
Supplement: Supplementary file 3 — Additional file 3. Protocol changes compared the original approved protocol. [file 13063_2022_6562_MOESM3_ESM.docx]

**Additional file 3.** Protocol changes compared the original approved protocol

| **Changes** | **Protocol Version 3.2** | **Protocol Version 3.3** | **Explanation** |
| --- | --- | --- | --- |
|  | Version date: 15^th^ of January 2020 | Version date: 15^th^ of April 2021 |  |
|  | Approval: 6^th^ of April 2020 | Approval: 28^th^ of April 2021 |  |
| Number of visits reduced | 7 regular study visits | 4 regular study visits, 1 optional study visit (wash-out) and 1 phone call with prior laboratory assessment at (external) location. | The run-in visit has been merged with the screening visit with X-LWK after confirmation of eligibility based on laboratory results and ECG.  The reduced number of study visits was a result of adaption to COVID-19 related restrictions and the wish to reduce visit burden to facilitate study participation. |
| Change of visit | First three visits (screening, run-in and baseline) and the last wash-out visit all with a 1 month interval | The first two visits (screening and baseline) and the optional last visit changed to a 3 months interval. For the baseline visit a more flexible planning is allowed (from 1 month interval after screening if preferred to even a 6 month interval in case of a eGFR > 25 ml/min/1.73m2.) | *Interval changed for convergence of regular outpatient clinic visits and to cover COVID-19 related study halts. |
| Change of definition of fasting blood withdrawal | At least 12h of fasting | At least 3h of fasting with request of only a light meal previous to fasting. | To facilitate study visits in the afternoon |
| Several changes in the  in- and exclusion criteria | eGFR inclusion range 10-45 ml/min/1.73m2 | Small shift of eGFR inclusion range to  15-50 ml/min/1.73m2. | Subjects with an eGFR range between 10-15 ml/min/1.73m2 have primary occupation with preparation to RRT and therefore not suitable for participation. Patients with an eGFR range between 45-50 ml/min/1.73m2 are situated within the same KDIGO stage 3 risk category and therefore appropriate for inclusion. |
|  | Subjects with the use of calcium carbonate excluded due to classification as phosphate binder | Subjects with the use of calcium carbonate included. | Frequently used in combination with Vitamin D and or as calcium supplement yet a negligible phosphate binding capacity in CKD stage 3 to 4 (contrary to its phosphate binding capacity in CKD stage 5). Reference: Hill, K. M., et al. (2013). "Oral calcium carbonate affects calcium but not phosphorus balance in stage 3-4 chronic kidney disease." Kidney International **83**(5): 959-966 |
|  | Subjects with atrial fibrillation or atrial flutter (AF) excluded | Only subjects with frequent AF or AF on recent ECG excluded. | Only AF during PWV measurement is problematic, but following this criteria unlikely to be present. |
|  | Participants with pacemaker excluded | Participants with pacemaker and ICD included | Decided after consultation with electrophysiologist that confirmed safety. And inclusion only after confirmation of correct R-top representation at ECG of PWV device. |
|  | Definition of bradycardia <60 bpm | Definition of bradycardia <50 bpm | Due to frequent and necessary beta-blocker use a bradycardia is common. With oral magnesium supplementation rapid shifts in electrolytes are not expected (contrary to dialysis) and therefore no relevant change is pulse is expected. Current oral magnesium intervention studies do not demonstrate data of concern with regards to bradycardia. |
|  | Participants with endoprothesis of the aorta excluded only from PET-scan | Full exclusion of participants with endoprothesis of the aorta | Because of influence on the validity of the primary endpoint of PWV and disrupted intervention effect due to the artificial material within the PWV measurement trajectory. |
| Additional  exclusion criteria |  | - Gastro-intestinal absorption disorders - Serious substance abuse - Recurrent incompliance of medication intake or hospital visits ‘no-shows’ - Insufficient understanding of the Dutch or English language |  |

*Abbreviations:* AF, atrial fibrillation and atrial flutter; bpm, beats per minute; ECG, electrocardiogram; eGFR, estimated glomerular filtration rate; h, hour; KDIGO, Kidney Disease Improving Global Outcomes (global nonprofit organization developing and implementing evidence-based clinical practice guidelines in kidney disease); PWV, pulse wave velocity; RRT, renal replacement therapy (dialysis or transplantation), X-LWK, X-Lumbale Wervel Kolom (an X-ray of the lower back displaying the abdominal aorta).*COVID-19 related protocol changes. In addition to these protocol changes this study also encountered many COVID-19 challenges. Examples include multiple study halts, repeated screening visits due to time passed, dropouts due to COVID-19 related concerns or declining renal function after initial consent, and reduced research support of the radiology department, pharmacy and clinical chemistry department due to their COVID-19 related research and clinical commitments
